# Valproate Attenuates Endoplasmic Reticulum Stress-Induced Apoptosis in SH-SY5Y Cells via the AKT/GSK3β Signaling Pathway

**DOI:** 10.3390/ijms18020315

**Published:** 2017-02-08

**Authors:** Zhengmao Li, Fenzan Wu, Xie Zhang, Yi Chai, Daqing Chen, Yuetao Yang, Kebin Xu, Jiayu Yin, Rui Li, Hongxue Shi, Zhouguang Wang, Xiaokun Li, Jian Xiao, Hongyu Zhang

**Affiliations:** 1 Key Laboratory of Biotechnology and Pharmaceutical Engineering, School of Pharmaceutical Sciences, Wenzhou Medical University, Wenzhou 325035, China; LiZM_YX@163.com (Z.L.); xukebin827@163.com (K.X.); pashayin@hotmail.com (J.Y.); xiaoerrui1989@163.com (R.L.); xue.henwuji@163.com (H.S.); huaikongwang@126.com (Z.W.); xiaokunli@163.com (X.L.); 2 Science and Education division, Cixi People’s Hospital, Wenzhou Medical University, Ningbo 315300, China; wufenzan@163.com; 3 Ningbo Medical Treatment Center, Li Huili Hospital, Ningbo 315000, China; rennie22@126.com; 4 Department of neurosurgery, The second Affiliated Hospital, Nanchang University, Nanchang 330006, China; lulpls@126.com; 5 Emergency Department, The Second Affiliated Hospital, Wenzhou Medical University, Wenzhou 325035, China; cdq1965@126.com (D.C.); yangyuetao1234@163.com (Y.Y.); 6 Institute of Life Sciences, Wenzhou University, Wenzhou 325035, China

**Keywords:** ER stress, valproate, apoptosis, neurological disorders, neurite outgrowth

## Abstract

Endoplasmic reticulum (ER) stress-induced apoptosis plays an important role in a range of neurological disorders, such as neurodegenerative diseases, spinal cord injury, and diabetic neuropathy. Valproate (VPA), a typical antiepileptic drug, is commonly used in the treatment of bipolar disorder and epilepsy. Recently, VPA has been reported to exert neurotrophic effects and promote neurite outgrowth, but its molecular mechanism is still unclear. In the present study, we investigated whether VPA inhibited ER stress and promoted neuroprotection and neuronal restoration in SH-SY5Y cells and in primary rat cortical neurons, respectively, upon exposure to thapsigargin (TG). In SH-SY5Y cells, cell viability was detected by the 3-(4,5-dimethyl-2-thiazolyl)-2,5-diphenyl-2-*H*-tetrazolium bromide (MTT) assay, and the expression of ER stress-related apoptotic proteins such as glucose‑regulated protein (GRP78), C/EBP homologous protein (CHOP), and cleaved caspase-12/-3 were analyzed with Western blot analyses and immunofluorescence assays. To explore the pathway involved in VPA-induced cell proliferation, we also examined p-AKT, GSK3β, p-JNK and MMP-9. Moreover, to detect the effect of VPA in primary cortical neurons, immunofluorescence staining of β-III tubulin and Anti-NeuN was analyzed in primary cultured neurons exposed to TG. Our results demonstrated that VPA administration improved cell viability in cells exposed to TG. In addition, VPA increased the levels of GRP78 and p-AKT and decreased the levels of ATF6, XBP-1, GSK3β, p-JNK and MMP-9. Furthermore, the levels of the ER stress-induced apoptosis response proteins CHOP, cleaved caspase-12 and cleaved caspase-3 were inhibited by VPA treatment. Meanwhile, VPA administration also increased the ratio of Bcl-2/Bax. Moreover, VPA can maintain neurite outgrowth of primary cortical neurons. Collectively, the neurotrophic effect of VPA is related to the inhibition of ER stress-induced apoptosis in SH-SY5Y cells and the maintenance of neuronal growth. Collectively, our results suggested a new approach for the therapeutic function of VPA in neurological disorders and neuroprotection.

## 1. Introduction

The endoplasmic reticulum (ER), an important subcellular organelle in eukaryotic cells, is the major site for protein folding, synthesis, trafficking and calcium storage, and it plays essential roles in multiple cellular processes that are required for cell survival and normal cellular functions [1]. Many external factors, such as oxidative stress, protein inclusion bodies, ischemia-reperfusion injury, spinal cord injury, disturbance of calcium homeostasis, and the inhibition of protein glycosylation, can disturb homeostatic ER function, leading to ER stress [2,3,4].

When the adaptive capacity of ER fails, the misfolded proteins accumulate in the ER lumen, and the unfolded protein response (UPR) is triggered [5]. ER stress triggers an evolutionarily conserved series of signal transduction events, such as enhancing the ability of proteins to fold properly, accelerating protein degradation, and increasing the probability of cell survival [6]. These signaling events aim to attenuate the accumulation of unfolded proteins in the ER. However, either under severe conditions or when ER stress is activated excessively, these events can induce cell death [7]. Several ER stress-associated transcription factors play important roles in regulating ER stress and any related apoptosis. Glucose-regulated protein 78 (GRP78), a member of the heat shock protein (HSP) family, is regarded as the marker of ER stress. It has been reported that GRP78 is released from IRE1 to support protein folding, and the expression GRP78 can be up-regulated by ER stress [8,9]. The activating transcription factors (ATF6) have been deemed to be a sensor of ER stress. When cleaved from the Golgi membrane, ATF6 enhances its localization to the nucleus, in which the transcription of UPR target genes were up-regulated, leading to apoptosis [10]. Activated IRE1 can promote the splicing of X-box-binding protein 1 (XBP-1) messenger RNA (mRNA), and mature XBP-1 promotes the transcription of UPR target genes such as C/EBP homologous protein (CHOP), leading to apoptosis [11]. UPR also results in intracellular calcium release, leading to cell death via caspase-12 and caspase-3 pathways [12]. Although ER stress has been demonstrated to play an important role in neuronal cell death, the correlative mechanism still requires further research.

Matrix metalloproteinase-9 (MMP-9) is involved in the stability of the extracellular matrix (ECM). In the brain, MMP-9 is critical for neuronal network remodeling and the integrity of the blood-brain barrier. One study reported that up-regulation of MMP-9 is related to the downstream ERK/JNK pathway [13]. c-Jun N-terminal kinases (JNKs) are a family of protein kinases that play an important role in many neurological disorders. JNKs are involved in apoptosis, neurodegeneration, cell differentiation and proliferation [14]. Mounting evidence suggests that the JNK pathway plays a major role in apoptosis in many cell death paradigms. It was reported that JNK can phosphorylate and directly activate apoptosis-related proteins such as BIM (homologous to BAX) [15]. Huili Zhu et al. reported that liraglutide exerts neuroprotection against ischemia-induced apoptosis through the activation of mitogen-activated protein kinase (MAPK) pathways as well as inhibition of the phosphorylation of c-Jun-N-terminal kinase (JNK) and p38 [16]. Whether Valproate (VPA) can also regulate the expression of MMP-9 and JNK still needs further study.

Valproate (VPA), a nonselective histone deacetylase inhibitor, is widely used for the treatment of seizures and bipolar mood disorder. Currently, it has been reported to exert a neuroprotective role in neurological disorders such as Amyotrophic lateral sclerosis (ALS), Parkinson’s disease (PD) and spinal cord injury (SCI) [17,18,19]. However, whether VPA can protect against apoptosis induced by ER stress (as well as the therapeutic mechanism involved) remains unclear. In addition, Zhang C et al. reported that VPA could be neuroprotective via activation of the extracellular signal-regulated kinase (ERK) and serine/threonine kinase (AKT) signaling pathways after traumatic brain injury (TBI) [20]. The phosphatidylinositol-3 kinase/protein kinase B (PI3K/AKT) signaling pathway is involved in the regulation of cell functions such as proliferation, differentiation, apoptosis, and glucose transport [21,22]. When AKT is phosphorylated, glycogen synthase kinase (GSK3β), which is a functional downstream target of AKT, is deactivated [23]. Whether VPA can regulate the AKT/GSK3β signaling pathway to exert a protection in SH-SY5Y cells is still unclear.

In the present study, we show that thapsigargin (TG) can cause apoptosis in SH-SY5Y cells via activation of ER stress. Moreover, we identify that VPA can attenuate TG-induced apoptosis in SH-SY5Y cells via suppression of ER stress-related proteins such as CHOP and caspase-12 and -3, activation of the AKT/GSK3β signaling pathway, and inhibition of JNK phosphorylation and MMP-9 expression. In addition, we show that TG can lead to the outgrowth stagnation of primary cortical neurons. However, VPA can maintain the development of neurons. Our findings demonstrate that VPA protects neurons against TG-induced apoptosis via inhibition of ER stress in SH-SY5Y cells, and against TG-induced outgrowth stagnation of primary cortical neurons. Thus, we provide new evidence for VPA in the treatment of neurological disorders.

## 2. Results

### 2.1. VPA Reduces TG-Induced Apoptosis in the SH-SY5Y Human Neuroblastoma Cell Line

**Figure 1:**
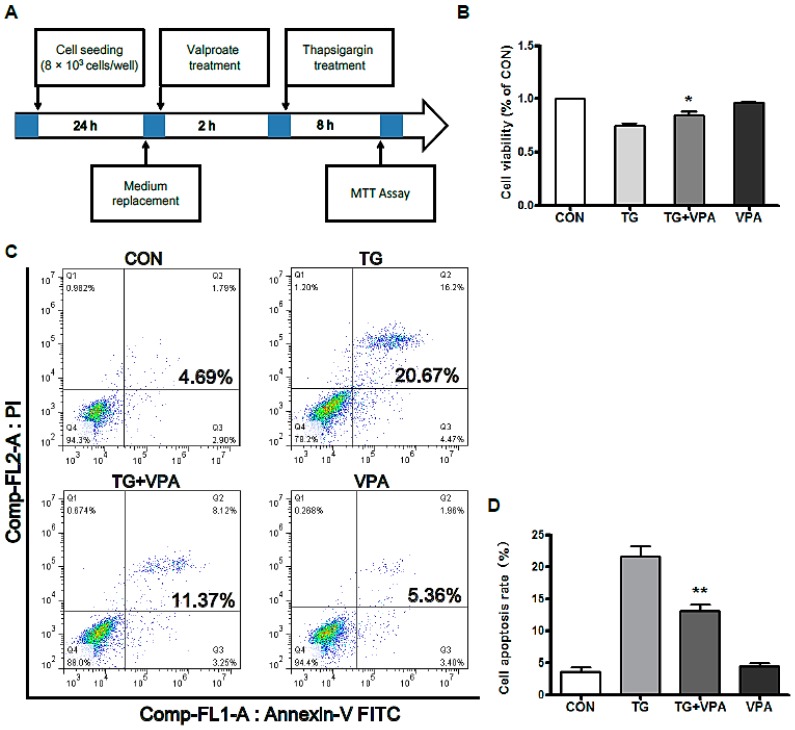
Valproate (VPA) reduces thapsigargin (TG)-induced apoptosis in SH-SY5Y cells. (**A**) The protocol of cell viability assay performed in this study. Cells were divided into four groups: the control group; the TG group; the TG and VPA group; the VPA group; (**B**) MTT assay result of VPA-treated SH-SY5Y cells exposed to TG. * *p* < 0.05 versus the TG group, *n* = 6; (**C**) Induction of apoptosis in human SH-SY5Y cells was determined by flow cytometry after treatment with TG, TG and VPA, and VPA; (**D**) The percentage of apoptotic cells in the treatment groups was calculated, ** *p* < 0.01 versus the control group, *n* = 3.

**Figure 2:**
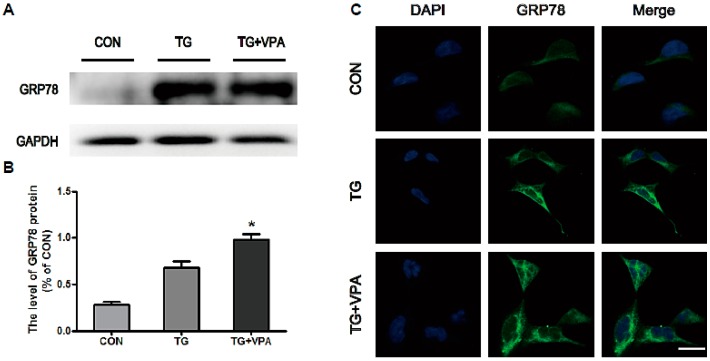
VPA promotes the expression of glucose-regulated protein (GRP78) in SH-SY5Y cells. (**A**,**B**) The protein level and the optical density of GRP78 in the control, TG, and TG and VPA groups. * *p* < 0.05 versus the TG group; (**C**) Immunofluorescence of GRP78. The nuclear are labeled by 4′,6-diamidino-2-phenylindole (DAPI) (**blue**), the target proteins (GRP78) are labeled by GFP-tagged proteins (**green**). Magnification was 40×. Data are the mean values ± SEM, *n* = 3.

Thapsigargin (TG) is a classic ER stress inducer that can initiate the stress condition in different cells [24]. When cells are exposed to TG, ER stress can be induced through inhibiting Ca2+-ATPase transporters at the ER membrane [25], which results in apoptosis when the degree of endoplasmic reticulum stress is overwhelming [26]. To determine whether VPA can inhibit cell apoptosis in SH-SY5Y cells treated with TG, the MTT assay was used as previously described [4]. After medium replacement, SH-SY5Y cells were treated with VPA 2 h prior to the administration of TG (Figure 1A). Compared with the control group, cell viability in the TG group was significantly decreased, while cell viability in the TG and VPA group was increased compared to the TG group, no change was seen in the VPA group compared to the control group (Figure 1B; *p* < 0.05). To investigate whether TG affects ER stress, we detected the expression of GRP78, the maker of ER stress. In the TG group, the expression of GRP78 was increased significantly. This result suggested that TG can efficiently cause ER stress. However, the expression of GRP78 was also increased in the TG and VPA group (Figure 2A,B; *p* < 0.05). Immunofluorescence microscopy was used to assess the expression of GRP78. Similar to the aforementioned results, the optical density of GRP78 was increased in the TG group, and the optical density of GRP78 in the TG and VPA group was also elevated (Figure 2C). These results suggest that VPA may maintain the degree of ER stress and enhance the ability of proteins to fold properly in the initial stage, thereby exerting a neuroprotection against cell death induced by TG in human SH-SY5Y cells.

### 2.2. VPA Attenuates TG-Induced ER Stress in SH-SY5Y Cells

**Figure 3:**
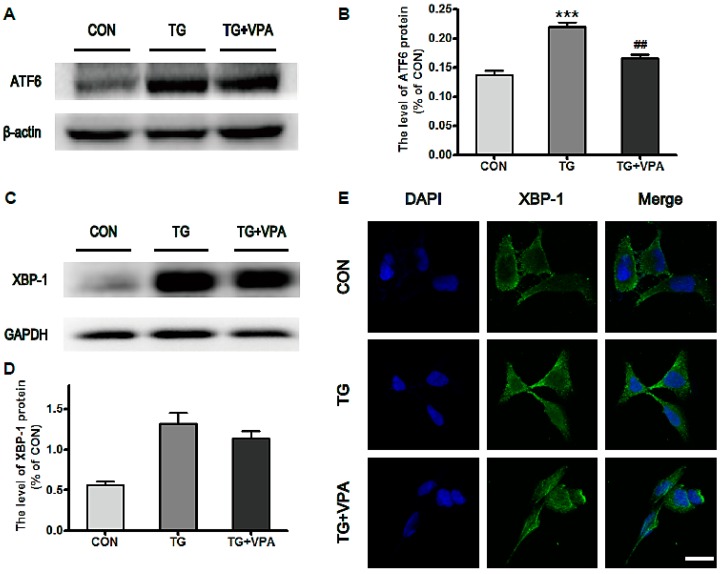
VPA attenuates TG induced ER stress in SH-SY5Y cells. (**A**,**C**) The protein level of ATF6 and XBP-1 in the control, TG, and TG and VPA groups; (**B**,**D**) The optical density analysis of ATF6 and XBP-1 protein. ^##^ *p* < 0.01 versus the TG group; *** *p* < 0.001 versus the control group; (**E**) Immunofluorescence of XBP-1 (green); the nuclear (blue) was labelled with DAPI. Magnification was 40×. Data are the mean values ± SEM, *n* = 3.

To evaluate the effect of VPA against ER stress induced by TG in SH-SY5Y cells, Western blot analysis was used to quantify the levels of the ER stress-associated proteins ATF6 and XBP-1. The levels of ATF6 and XBP-1 were significantly increased in the TG group compared to the control group. In the TG and VPA group, the levels of ATF6 (*p* < 0.01) and XBP-1 were reduced compared to the TG group (Figure 3A–D). Immunofluorescence microscopy was used to assess the level of XBP-1. Similar to the Western blot results, the optical density of XBP-1 was up-regulated in the TG group. However, compared with the TG group, the optical density of XBP-1 in the TG and VPA group was down-regulated (Figure 3E). These results demonstrated that VPA can efficiently reduce ER stress induced by TG in SH-SY5Y cells.

### 2.3. VPA Inhibits the Expression of C/EBP Homologous Protein (CHOP) and Caspase-12 in TG-Induced SH-SY5Y Cells

**Figure 4:**
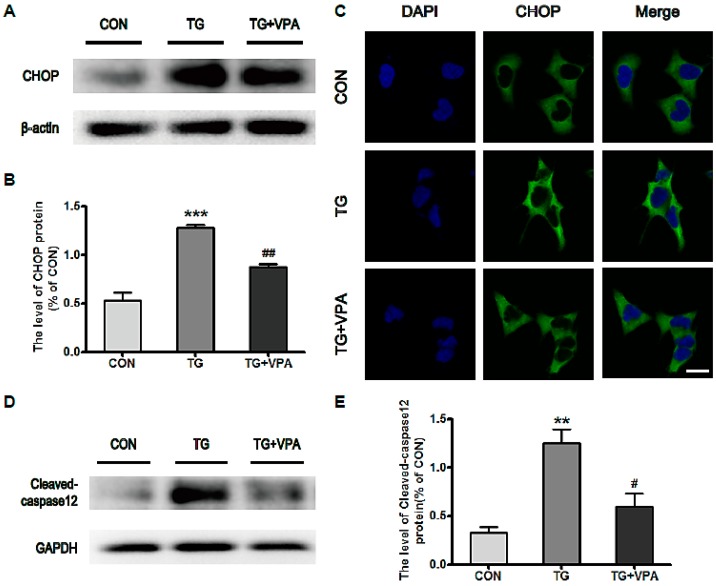
VPA inhibits ER stress-induced apoptosis in SH-SY5Y cells. (**A**,**D**) The level of CHOP and cleaved-caspase12 in the control, TG, and TG and VPA groups. *n* = 5; (**B**,**E**) Densitometric analysis of CHOP and cleaved caspase-12, ^#^ *p* < 0.05, ^##^ *p* < 0.01 versus the TG group; ** *p* < 0.01, *** *p* < 0.001 versus the control group; (**C**) Immunofluorescence of CHOP (**green**); the nuclear (**blue**) was labelled with DAPI. Magnification was 40×. Data are the mean values ± SEM, *n* = 3.

Next we investigated the molecular mechanism of ER stress-induced apoptosis in SH-SY5Y cells by using a Western blot assay to detect the expression of CHOP and cleaved caspase-12. It was found that the expression levels of CHOP and cleaved caspase-12 were increased in TG-treated cells. Compared with the TG group, the expression levels of CHOP (*p* < 0.01) and cleaved caspase-12 (*p* < 0.05) were significantly reduced (Figure 4A–E). Likewise, the optical density of CHOP was increased significantly in the TG group compared to the control group, but in the TG and VPA group, the expression of CHOP was decreased after treatment with VPA (Figure 4C). These results suggested that VPA can reduce ER stress-induced apoptosis through the inhibition of CHOP and caspase-12 expression in SH-SY5Y cells.

### 2.4. VPA Inhibits ER Stress-Induced Apoptosis and Up-Regulates the Ratio of Bcl-2/Bax in SH-SY5Y Cells

**Figure 5:**
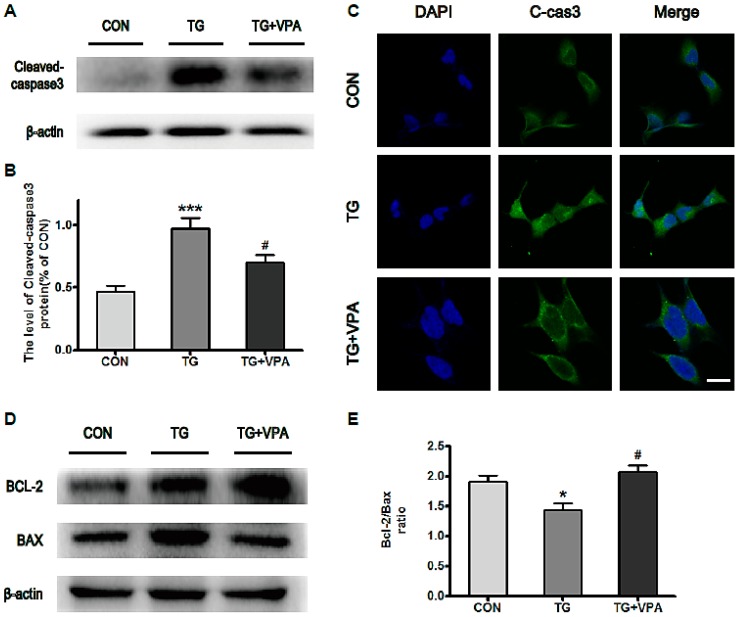
VPA reduces the level of cleaved caspase-3 and increases the ratio of Bcl-2/BAX in SH-SY5Y cells. (**A**,**B**) The expression of cleaved caspase-3 in the three groups was investigated by Western blot analysis and immunofluorescence assay. ^#^ *p* < 0.05 versus the TG group; *** *p* < 0.001 versus the control group; (**C**) Immunofluorescence of cleaved-caspase-3 (green); the nuclear (blue) was labelled with DAPI. Magnification was 40×; (**D**) Protein levels of Bcl-2 and Bax; (**E**) The ratio of Bcl-2/Bax. * *p* < 0.05 versus the control group. ^#^ *p* < 0.05 versus the TG group (*t*-test). Data are the mean values ± SEM, *n* = 4.

We next detected the level of cleaved caspase-3. We found that the level of cleaved caspase-3 was enhanced when cells were exposed to TG for varying amounts of time. By contrast, in the TG and VPA group, the level of cleaved caspase-3 was reduced (Figure 5A,B; *p* < 0.05). An immunofluorescence assay detecting the level of caspase-3 elicited the same result (Figure 5C). Meanwhile, we examined the levels of the anti-apoptotic protein Bcl-2 and pro-apoptotic protein Bax via Western blot. The results illustrated that VPA down-regulated the levels of Bcl-2 but up-regulated the levels of Bax (Figure 5D). Comprehensively, the ratio of Bcl-2/Bax was increased in cells pretreated with VPA (Figure 5E; *p* < 0.05). These results revealed that VPA can protect neuronal cells against apoptosis induced by ER stress.

### 2.5. VPA Promotes Cell Proliferation through the PI3K/AKT/GSK3β Signaling Pathway in SH-SY5Y Cells

**Figure 6:**
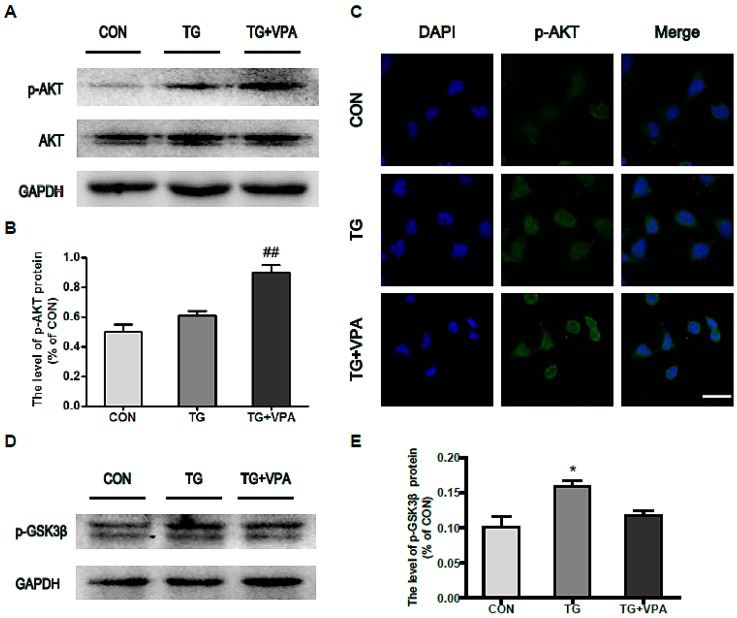
VPA activates the PI3K/Akt/GSK3β signaling pathway in SH-SY5Y cells. (**A**) Protein level of p-AKT in the control, TG, and TG and VPA groups. *n* = 3; (**B**) The optical density analysis of p-AKT, ^##^ *p* < 0.01 versus the TG group; (**C**) Immunofluorescence of p-AKT (**green**); the nuclear (**blue**) was labelled with DAPI. Magnification was 40×; (**D**) Protein level of p-GSK3β after VPA treatment; (**E**) Densitometric analyses of p-GSK3β, * *p* < 0.05 versus the control group. Data are the mean values ± SEM, *n* = 3.

To investigate the related signaling pathways in which VPA promotes cell proliferation, we examined the phosphorylation status both of AKT and GSK3β in SH-SY5Y cells and found that VPA can increase the phosphorylation of AKT (*p* < 0.01) but decrease the expression of p-GSK3β (Figure 6A,B,D,E). An immunofluorescence assay detecting the phosphorylation status of AKT showed the same results as the Western blot (Figure 6C). These results suggest that VPA-promoted cell proliferation is related to the activation of the PI3K/AKT/GSK3β signaling pathway in SH-SY5Y cells.

### 2.6. VPA Prevents Cell Apoptosis by Inhibiting MMP-9 Expression and c-Jun N-Terminal Kinase (JNK) Phosphorylation

**Figure 7:**
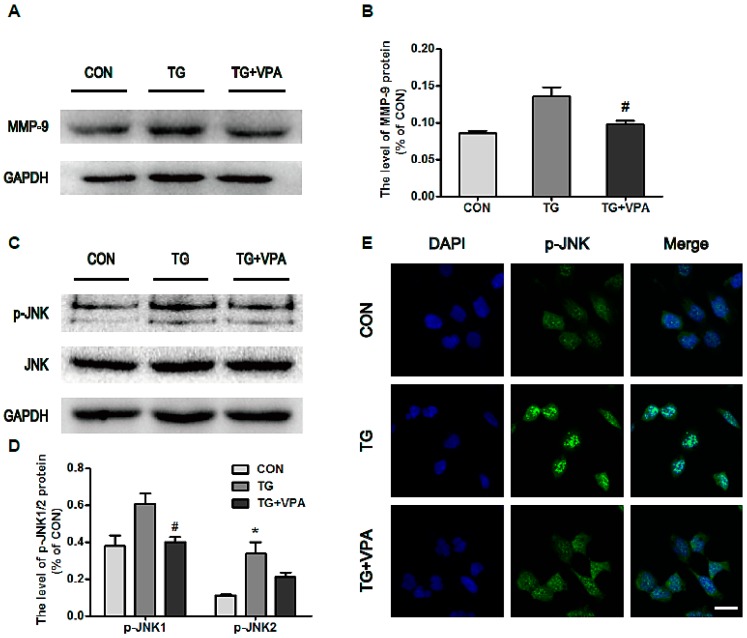
VPA inhibits the expression of MMP-9 and the phosphorylation of JNK. (**A**) Protein level of MMP-9 after VPA treatment; (**B**) Densitometric analyses of MMP-9, ^#^ *p* < 0.05 versus the TG group; (**C**) Protein level of p-JNK1/2 in the control, TG, and TG and VPA groups. *n* = 3; (**D**) The optical density analysis of p-JNK1/2, ^#^ *p* < 0.05 versus the TG group, * *p* < 0.05 versus the control group; (**E**) Immunofluorescence of p-JNK (green) and the nuclear (blue). Magnification was 40×. Data are the mean values ± SEM, *n* = 3.

**Figure 8:**
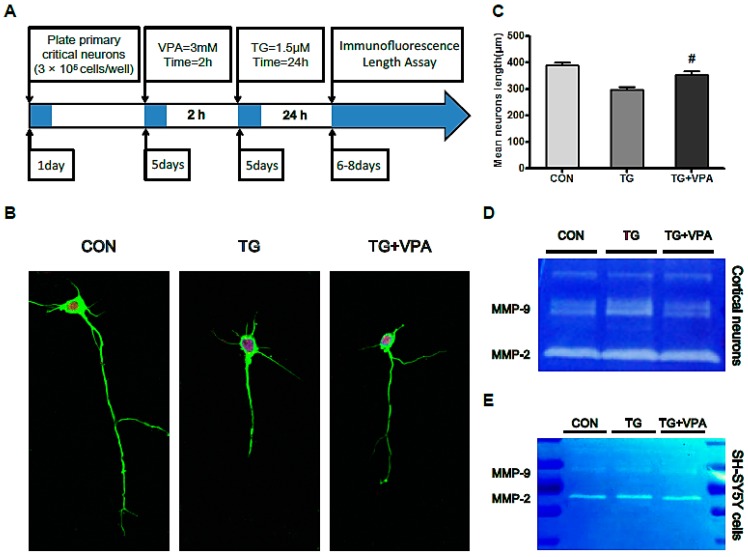
VPA plays a neuroprotection role in primary cortical neurons and affects matrix metalloproteinase (MMP) family protein activation. (**A**) The protocol of the experiment in primary cortical neurons; (**B**) Immunofluorescence of primary cortical neurons after TG treatment. β-IIItublin (**green**); NeuN (**red**); the nuclear was labeled by DAPI (**blue**). Magnification was 60×; (**C**) The mean neurite length of primary cortical neurons, ^#^ *p* < 0.05 versus the TG group; (**D**,**E**) Gelatin zymography for the cell medium in primary cortical neurons and neuronal cells line SH-SY5Y. Data are the mean values ± SEM, *n* = 3.

To assess the underlying mechanism of VPA in TG-induced apoptosis, we detected the expression of MMP-9 and the phosphorylation status of JNK. We found that TG can up-regulate both the expression of MMP-9 and the phosphorylation of JNK. Pretreatment with VPA showed down-regulation of both of these changes (Figure 7A,B,D,E; *p* < 0.05), and the analysis of gelatin zymography showed that MMP-9 was also down-regulated by VPA, but MMP-2 was unchanged (Figure 8D,E) in primary cortical neurons and in SH-SY5Y cells . These results indicated that VPA may regulate the level of MMP-9 only, not the level of MMP-2 both in primary cortical neurons and in SH-SY5Y cells. They suggest that the neuroprotection VPA exhibits in cortical neurons and SH-SY5Y cells may be related to the ability to block the activity of MMP-9 and prevent MMP-9 from degrading the extracellular matrix and thereby stabilize cells when cells are exposed to TG. The immunofluorescence levels of p-JNK also suggested the inhibitory effect of VPA on TG-induced JNK phosphorylation (Figure 7C). These results suggest that the anti-apoptosis effect of VPA may be related not only to the inhibition of CHOP and caspase-12/-3 but also to the inhibition of MMP-9 and phosphorylation of JNK.

### 2.7. VPA Contributes to Axonal Maintenance in Primary Critical Neurons Treated with TG

Prior research has illustrated that VPA exerts a protective effect in SH-SY5Y cells. To further validate the effect of VPA in primary neurons, we detected the neurite length of primary critical neurons after the administration of TG and VPA by immunofluorescence staining with β-III tubulin and Anti-NeuN. Our results showed that the mean length of neurites treated with TG was 299.3 ± 25.7 µm, which was obviously increased upon pretreatment with VPA (355.4 ± 34.2 µm, *p* < 0.05) (Figure 8A–C). These data suggested that the ER stress inducer TG inhibited neurite outgrowth and that VPA protected primary cortical neurons against the toxicity of TG.

## 3. Discussion

Central nervous system (CNS) diseases such as Parkinson’s disease (PD), Alzheimer’s disease (AD), amyotrophic lateral sclerosis (ALS) and cerebral ischemia are involved in the activation of ER stress. The generation of impaired mitochondrial ATP causes secondary ER failure and the accumulation of aggregated synuclein in the ER, which is regarded as the main mechanism of ER dysfunction in PD [27]. For AD, the impairment of ER calcium homoeostasis induced by the direct toxic effects of β-amyloid is the proposed mechanism of ER dysfunction [28]. Meanwhile, the accumulation of mutant SOD1 in the ER may also induce amyotrophic lateral sclerosis [29]. Regarding cerebral ischemia, one of the classic histopathological characteristics is energy deficiency that results in the dysfunction of ER chaperones and enzymes [30]. All of these studies have demonstrated that the regulation of ER stress is an important strategy to rescue these CNS diseases. In addition, there are reports that ER dysfunction has been linked to impaired synaptic plasticity and to the pathophysiology of mental disorders such as bipolar disorder (BD) [31,32]. In clinical applications, VPA is traditionally used to treat BD. Recently, it has been reported that VPA protects normal hippocampal neurons from radiation-induced cytotoxicity both in vitro and in vivo [33] and enhances the developmental competence of bovine SCNT embryos by up-regulating the level of the ER stress-associated protein GRP78 [34]. However, the exact mechanism of how VPA regulates ER stress requires additional research.

TG treatment is often used as an ER stress initiation agent in vitro. GRP78, a heat shock protein present in all cells, has been widely used as a marker for ER stress [35,36]. It was reported that propofol could inhibit GRP78 expression in TG-treated ARPE-19 cells [37]. However, in our study, VPA promoted the expression of GRP78 (Figure 1C). Similarly, Sofia Esteves et al. reported that chronic VPA treatment increases GRP78 protein levels in the cerebellums of CMVMJD135 mice [38]. The role of these two drugs on the GRP78 may be different, and the specific mechanism involved in GRP78 regulation requires further exploration. Lu T et al. reported that isoquercetin (Iso) down-regulated the mRNA levels of ER stress genes such as C/EBP homologous protein (CHOP) and cleaved caspase-12 in tunicamycin (TUN)-induced DRG neurons [39]. In the present study, we detected the expression of CHOP and cleaved caspase-12 and found that the levels of the ER stress‑associated proteins CHOP and cleaved caspase-12 were all significantly increased in the TG group (Figure 3A,D). This suggests that apoptosis in SH-SY5Y cells can be activated via an ER stress‑induced pathway. The report by Jee Y. Lee et al. showed that VPA inhibited the activation of caspase-3 after spinal cord injury [40]. In addition, it was reported that in a mouse model of ALS, VPA was shown to up-regulate Bcl-2 mRNA levels in the spinal cord [41]. Additionally, the elevated Bcl-2/Bax ratio illustrated that VPA has some ulterior profitable effect on ER stress. In this study, our data also showed that the activation of caspase-3 was inhibited in the TG and VPA group (Figure 4A), and the ratio of Bcl-2/Bax was evaluated in SH-SY5Y cells (Figure 4D). This result suggested that the effect of VPA against ER stress occurs by affecting caspase-3 and up-regulating the ratio of Bcl-2/Bax. Based on the aforementioned results, the effect of VPA against ER stress-induced apoptosis is not only promoting the expression of GRP78 and the anti-apoptotic protein Bcl-2 but also inhibiting the activity of caspase-12 and caspase-3 as well as down-regulating CHOP and the pro-apoptotic protein Bax.

Furthermore, the effects of VPA on cell proliferation, apoptosis, metastasis and invasion were evaluated. AKT is a serine/threonine-specific protein kinase and is also known as protein kinase B (PKB). AKT participates in multiple cellular processes, especially in the modulation of cell death and survival [42]. GSK3β, a serine/threonine protein kinase, was originally discovered in the context of its involvement in regulating glycogen synthase. GSK3β is also integrally tied to the pathways of cell proliferation and apoptosis [43]. In addition to its inhibitory effect on ER stress, VPA was reported to markedly up-regulate AKT expression in a rat traumatic brain injury model [20]. Chu, T et al. reported that VPA can counteract secondary damage to functionally rescue SCI in animal models by improving neuroprotection and neurogenesis via inhibition of GSK3β [44]. The correlation between VPA and the AKT and GSK3β pathways also was investigated in our present study. VPA treatment up-regulated the phosphorylation of AKT and inhibited the expression of GSK3β. This finding suggests that the neuroprotective effects of VPA are also mediated through the activation of the AKT/GSK3β signaling pathway.

The MMP family comprises a type of zinc-dependent endopeptidase, and its principal function is to degrade proteins in the matrix. Ying, G. Y. et al. reported that 300 mg/kg of VPA can effectively decrease the expression and activity of MMP-9 as well as reduce claudin-5 and occludin degradation after subarachnoid hemorrhage (SAH) in rats [45]. In addition, Xia Zhao et al. reported that VPA can inhibit MMP-2 and MMP-9 protein expression in HepG2 cells [46]. To estimate the effect of VPA on the migration and invasion of SH-SY5Y cells, we tested the protein levels of MMP-9 and the enzyme activity of MMP-9/-2 in gelatin. We found that VPA down-regulated the expression of MMP-9 (Figure 7A), but gelatin zymography for cell medium after TG and VPA treatment showed that MMP-2 presented no significant changes in activity (Figure 8D). From this result we concluded that VPA can inhibit the migration and invasion of SH-SY5Y cells and speculated that MMP-9 may be the main target of VPA in SH-SY5Y cells. On the other hand, Xiang Yin et al. reported that norepinephrine (NE) enhances LPS-induced MMP-9 expression through β-adrenergic receptors and the downstream ERK/JNK-c-Fos pathway in human THP-1 cells [13]. Similarly, we also examined the expression of JNK in SH-SY5Y cells. We found that the phosphorylation of JNK was increased when cells were exposed to TG but decreased after the administration of VPA. This result seems to be inconsistent with some previous reports. Kim J.N. et al. reported that VPA regulates α-synuclein expression through up-regulating the phosphorylation of JNK in rat primary astrocytes [47]. V. Singh et al. found that LPS treatment of microglia leads to the activation of p38 and the MAPKs ERK1/2 and JNK. However, the treatment of microglia with VPA (0.5 and 1 mM) did not change LPS-induced activation of these protein kinases [48]. The specific mechanisms involved in this activity are still unexplored.

Furthermore, there are many studies suggesting that VPA exerts neurotrophic properties against a series of insults. It was reported that the loss of dopamine neurons in rotenone-lesioned animals was blocked by chronic VPA treatment [49]. Zhang Z. et al. reported that 300 mg/kg of VPA markedly attenuated I/R-induced retinal neuron apoptosis, damage to retinal ganglion cells, and morphological injury to the retina and optic nerve axons [50]. To further confirm the neuroprotection of VPA, we used TG to focus on primary cortical neurons. After treatment with TG, the morphology of the neurons was incomplete, and the axon length was significantly shorter compared to the control group. After treatment with VPA, the length of the axons was increased but was still shorter than the control group (Figure 8B,C). These results show that VPA can effectively protect neurons, which is consistent with the results reported previously in the literature.

## 4. Materials and Methods

### 4.1. Primary Cortical Neurons Culture

Adult Sprague-Dawley (SD) rats were obtained from the Animal Center of the Chinese Academy of Science (Shanghai, China). All experimental procedures were approved by the Laboratory Animal Ethics Committee of Wenzhou Medical University (SYXK2014-0006) and were performed in accordance with the Guide for the Care and Use of Laboratory Animals. Primary cortical neurons were established from the brains of neonatal Sprague-Dawley rats (<3 days of age). Cortex was separated from the brains and rinsed in ice-cold dissection buffer. After removing the blood vessels, tissues were treated with 0.25% trypsinin Hank’s balanced salt solution for 10 min at 37 °C. The whole solution was filtered through stainless steel (200 mesh; BD Biosciences, San Jose, CA, USA). Cell suspension was centrifuged twice at 1000 rpm for 5 min and the cell pellets were resuspended in DMEM/F-12 with 10% fetal bovine serum, 100 U/mL penicillin, 100 mg/L streptomycin and 0.5 mM glutamine. Cells were seeded at a density of 3 × 10^5^/mL in 6-well plates kept at 37 °C in a 5% CO_2_ incubator. After 4 h, the culture medium was changed to Neurobasal Medium (Gibco, life technologies) with 2% B27 and changed every 2–3 days. To prevent the growth of non-neuronal cells, arabinosylcytosin (10 mg/L; Sigma-Aldrich, St. Louis, MO, USA) was added at 36 h. After 12 h, the culture medium was changed to neurobasal medium. At 5 days after seeding, the primary neuron was treated with 1.5 µM TG for 24 h, and dealt with fluorescent staining. Then the length of axon was detected by Image J (version 1.48) software. The experiment was performed at 5–7 days after seeding.

### 4.2. Cell Culture

SH-SY5Y cells were purchased from the China Center for Type Culture Collection (Wuhan University, China, 22-4-2015, http://www.cctcc.org) and cultured in Dulbecco’s Modified Eagle’s medium (DMEM, Invitrogen, Carlsbad, CA, USA) containing 10% Fetal Bovine Serum (FBS, Invitrogen), and antibiotics (100 units/mL penicillin and100 µg/mL streptomycin) incubated in a humidified atmosphere containing 5% CO_2_ at 37 °C. Cells were passaged every 3–5 days with 0.25% trypsin-EDTA (Invitrogen, Carlsbad, CA, USA).

### 4.3. Cell Viability Assay

To determine the effect of VPA against TG (Sigma-Aldrich)-induced apoptosis, SH-SY5Y cells were seeded on 96-well plates (8 × 10^3^ cells/well) and incubated in a humidified atmosphere containing 5% CO_2_ at 37 °C for 24 h prior to treatment. After replacing the entire medium with fresh medium, each cell was treated with different concentrations of an ER stress activator, various dosages of TG (0, 1, 2.5, 5, and 10 µM) for 8 h. To determine the effect of VPA (Sigma-Aldrich), VPA (3 mM) was added 2 h prior to the addition of TG. Cell viability was detected by MTT (3-(4,5-dimethyl-2-thiazolyl)-2,5-diphenyl-2-*H*-tetrazolium bromide) assays. 20 µL MTT (5 mg/mL in PBS) was add to the cells for 4 h. Then cells were washed with PBS (pH 7.4), and 150 µL DMSO was used to solubilize the formazan crystals. Fluorescence intensity was measured at 595 nm. Optimal conditions of 10 µM TG and 3 mM VPA were used for the subsequent experiments.

### 4.4. Cell Apoptosis Assay

SH-SY5Y cells were plated on 60-mm plates for 12 h, and then treated with TG (10 µM) and VPA (3 mM) for 8 h. Cells were then harvested, washed twice with ice-cold PBS, and detected for apoptosis by double staining with fluorescein isothiocyanate (FITC) conjugated Annexin V and propidium iodide (PI) in a binding buffer for 15 min using a FACSC alibur flow cytometer (BD Biosciences).

### 4.5. Western Blot Analysis

SH-SY5Y cells were lysed using RIPA buffer (25 mM Tris-HCl, 150 mM NaCl, 1% Nonidet P-40, 1% sodium deoxycholate, and 0.1% SDS) with protease inhibitors (Sigma-Aldrich) and phosphatase inhibitors (Sigma-Aldrich). The samples above were quantified with bicinchoninic acid (BCA) reagents (Thermo, Rockford, IL, USA) and heated in a loading buffer. Proteins (50 µg) were separated on a 12% gel and transferred onto a PVDF membrane (Bio-Rad, Hercules, CA, USA). The membranes were blocked with 5% non-fat milk (Bio-Rad) in TBST (10 mM Tris-HCl, (pH 7.6), 100 mM NaCl and 0.1% Tween 20) for 2 h at room temperature and incubated with the desired primary antibody: GRP78 (1:300, dilution, Santa Cruz Biotechnology, Santa Cruz, CA, USA), ATF6 (1:1000, dilution, Abcam, UK), XBP-1 (1:300, dilution, Santa Cruz Biotechnology), CHOP (1:300, dilution, Santa Cruz Biotechnology), BCL-2 (1:300, dilution, Santa Cruz Biotechnology), BAX (1:300, dilution, Santa Cruz Biotechnology), cleaved caspase-12 (1:1000, dilution, Santa Cruz Biotechnology), or cleaved caspase-3 (1:1000, dilution, Abcam) in 5% milk in TBS + Tween overnight. The membranes were washed with TBST 3 times and incubated with horseradish peroxidase-conjugated secondary antibodies (goat anti-mouse and goat anti-rabbit (Pierce Biotechnology, Rockford, IL, USA)) for 1.5 h at room temperature. Immunoreactive bands were visualized by ChemiDicTM XRS + Imaging System (Bio-Rad), and the band densities were quantified with Multi Gauge Software of Science Lab 2006 (FUJIFILM Corporation, Tokyo, Japan). The relative densities of the bands were analyzed with Quantity One (version 4.5.2; Bio-Rad). Quantities of band densities were normalized using GAPDH and β-actin.

### 4.6. Immunofluorescence Assay

SH-SY5Y cells inoculated on coverslips were cultured for 24 h. Then, the cell was fixed with phosphate buffer solution (PBS) containing 4% paraformaldehyde for 10 min, and permeabilized with 0.25% Triton X-100 for 30 min. Following blocking with 5% BSA in PBS for 1 h, cells were incubated with different targets: anti-GRP78 (1:500, dilution, Abcam); anti-CHOP (1:500, dilution, Abcam); anti-XBP-1 (1:500, dilution, Abcam); or anti-caspase-3 (1:500, dilution, Abcam) overnight at 4 °C. After primary antibody incubation, cells were washed with PBS for 3 × 10 min at room temperature, and incubated with Alexa-Fluor488/594 donkey anti-rabbit/mouse secondary antibody (1:1000, dilution, Abcam) for 1 h at room temperature, stained with DAPI (2 µg/mL) for 5 min and washed with PBS for 3 × 5 min at room temperature. The fluorescent images were taken by Nikon confocal laser microscope (Nikon, A1 PLUS, Tokyo, Japan).

### 4.7. Gelatin Zymography

The activity of MMP-2 and -9 was examined by gelatin zymography. Briefly, the protein concentration of mediums was determined by the bicinchoninic acid method (BCA protein assay kit; Thermo Fisher Scientific, Rockford, IL, USA). After detecting the concentration of protein, equal amounts of protein (30 µg)were loaded on a Novex 10% zymogram gel (EC61752; Invitrogen) and separated by electrophoresis with 100 V (19 mA) at 4 °C for 6 h. Gel was incubated with renaturing buffer (2.5% Triton X-100) at 15–30 °C for 1 h to restore the gelatinolytic activity of the proteins, and then incubated for 24 h in a developing buffer, including Tris 50 mM/L (pH 7.6), CaCl_2_ 25 mM, NaCl 0.2 mM, and 0.02% (*w*/*v*) Brij-35 (Sigma-Aldrich) at 37 °C. Next, the gel was stained with 0.5% Coomassie blue for 60 min and then faded with 40% methanol containing 10% acetic acid until the appropriate color contrast was achieved. The relative intensity of zymography (relative to sham or vehicle) was measured and analyzed by AlphaImager (version 1.0) software (α Innotech Corporation, San Leandro, CA, USA). The background was subtracted from the optical density measurements. Experiments were repeated three times and the values obtained for the relative intensity were subjected to statistical analysis.

### 4.8. Statistical Analysis

Data were expressed as mean ± SEM. The statistical significance between two groups and multiple groups were determined by the Student’s *t*-test and One-way Analysis-of-variance (ANOVA) test, followed by Dunnett’s post hoc test with the values *p* < 0.05 considered significant.

## 5. Conclusions

Our results provide evidence that the mood stabilizer valproic acid has anti-apoptotic and neuroprotection qualities in SH-SY5Y cells and primary cortical neurons. VPA significantly inhibited ER stress and up-regulated the activity of AKT expression. The JNK, MMP-9, and GSK3β signaling pathways were all involved in VPA-induced neuroprotection. Because of its pleiotropic effects, VPA is a particularly important therapeutic candidate for the clinical treatment of neurodegenerative diseases, which requires further study.
